# Determinants of tuberculosis transmission and treatment abandonment in Fortaleza, Brazil

**DOI:** 10.1186/s12889-017-4435-0

**Published:** 2017-05-25

**Authors:** Guy Harling, Antonio S. Lima Neto, Geziel S. Sousa, Marcia M. T. Machado, Marcia C. Castro

**Affiliations:** 1 Department of Global Health and Population, Harvard T.H. Chan School of Public Health, 665 Huntington Avenue, Building I, Room 1113, Boston, MA 02115 USA; 2 Research Department of Infection and Population Health, University College London, London, UK; 3 Fortaleza Municipal Health Secretariat (SMS-Fortaleza), Fortaleza, CE Brazil; 4 University of Fortaleza (UNIFOR), Fortaleza, CE Brazil; 5 Federal University of Ceará (UFC), Fortaleza, CE Brazil

**Keywords:** Fortaleza, Brazil, Tuberculosis, Treatment, Treatment failure, Spatial analysis, Social determinants, Epidemiology

## Abstract

**Background:**

Tuberculosis (TB) remains a public health problem, despite recent achievements in reducing incidence and mortality rates. In Brazil, these achievements were above the worldwide average, but marked by large regional heterogeneities. In Fortaleza (5th largest city in Brazil), the tuberculosis cure rate has been declining and treatment abandonment has been increasing in the past decade, despite a reduction in incidence and an increase in directly observed therapy (DOT). These trends put efforts to eliminate tuberculosis at risk. We therefore sought to determine social and programmatic determinants of tuberculosis incidence and treatment abandonment in Fortaleza.

**Methods:**

We analyzed sociodemographic and clinical data for all new tuberculosis cases notified in the Notifiable Diseases Information System (SINAN) from Fortaleza between 2007 and 2014. We calculated incidence rates for 117 neighborhoods in Fortaleza, assessed their spatial clustering, and used spatial regression models to quantify associations between neighborhood-level covariates and incidence rates. We used hierarchical logistic regression models to evaluate how individual- and neighborhood-level covariates predicted tuberculosis treatment abandonment.

**Results:**

There were 12,338 new cases reported during the study period. Case rates across neighborhoods were significantly positively clustered in two low-income areas close to the city center. In an adjusted model, tuberculosis rates were significantly higher in neighborhoods with lower literacy, higher sewerage access and homicide rates, and a greater proportion of self-reported black residents. Treatment was abandoned in 1901 cases (15.4%), a rate that rose by 71% between 2007 and 2014. Abandonment was significantly associated with many individual sociodemographic and clinical factors. Notably, being recommended for DOT was protective for those who completed DOT, but associated with abandonment for those who did not.

**Conclusion:**

Low socioeconomic status areas have higher tuberculosis rates, and low socioeconomic individuals have higher risk of treatment abandonment, in Fortaleza. Treatment abandonment rates are growing despite the advent of universal DOT recommendations in Brazil. Proactive social policies, and active contact tracing to find missed cases, may help reduce the tuberculosis burden in this setting.

**Electronic supplementary material:**

The online version of this article (doi:10.1186/s12889-017-4435-0) contains supplementary material, which is available to authorized users.

## Background

Tuberculosis (TB) disease remains a major public health problem, with almost 10 million cases and 1.5 million deaths in 2014, making it the leading infectious cause of death worldwide [1]. Nevertheless, considerable global progress has been made in reducing TB mortality. The Millennium Development Goal (MDG) for TB, to halt and reverse global incidence, has been achieved with a 40% fall in mortality, and an 18% fall in incidence, rates since 2000 [2]. The World Health Organization (WHO) has highlighted that achieving the vision of ending the global TB epidemic by 2035 will require consideration of, and action on, a wide range of risk factors including clinical, social and structural items [3–5].

Brazil remains one of 22 high TB ​​burden countries (HBCs) that jointly account for 80% of the global burden of disease [2]. It was, however, one of nine HBCs to meet all three TB reduction targets for 2015 established for the MDGs: between 1990 and 2014 the Brazilian TB incidence rate declined from 51.8 to 33.2 cases per 100,000, and both prevalence and mortality rates fell by more than 50% [6]. While these decreases were greater than those observed worldwide, they were not homogenous across the country [6, 7]. In 2015, Ceará had the seventh highest pulmonary TB incidence rate out of the 27 states in Brazil [8]. Despite a slight decrease in TB incidence rates in recent years, cure rates fell from 86% in 2007 to 68% in 2013, more than 15% below the minimum level recommended by WHO. Treatment abandonment (non-completion not due to healthcare indication) also increased from 7.5% to 11.4% [9].

Within Ceará, its capital Fortaleza has extremely high TB infection risk, documenting 1526 new cases in 2013 (an incidence rate of 59.8 per 100,000). These new cases reported a total of 7090 contacts, but only 2348 (33.1%) were traced by public health workers. Contact tracing has been worsening in Ceará in the recent past [9]. Between 2007 and 2014, TB incidence rate fell from 65 to 59 cases per 100,000 in 2014, however the cure rate fell from 74% to 54%, and the abandonment rate is approaching 20% [10].

To reduce treatment abandonment, increase treatment success, and reduce cases of multidrug-resistant TB, the Brazilian government recommends the use of directly observed treatment (DOT) for all confirmed cases [11], where DOT is defined as daily observation of treatment from Monday to Friday each week [12]. In Fortaleza, DOT is performed through a mixture of clinic-, home- and work-based observation by community health workers and other health professionals (with support from family and friends). However, limited resources prevented the achievement of this recommendation; in Fortaleza 62% of all TB cases were recommended for DOT between 2007 and 2014). This rate rose over time, even though official government policy did not change in this period.

The active search for residents with respiratory symptoms by community health workers also constitutes a powerful weapon for breaking the chain of transmission. Sputum smear-positive TB cases require immediate examination of household contacts in order to find new cases. Indeed, low rates of detection of smear-positive cases are associated with increasing trends in national TB incidence in Latin American and Caribbean countries [13]. The slow onset and chronic nature of TB can delay infected individuals seeking care, therefore active surveillance strategies are recommended in high transmission areas.

Fortaleza has many primary health care units able to diagnose and treat TB patients. However, there is a shortage of primary care professionals, and the Family Health Strategy (FHS), used for delivering primary care, covered only 55% of the city’s population in 2013 [14]. In 2013, only 38% of new cases in Fortaleza maintained DOT throughout treatment [7].

Fortaleza’s high incidence combined with low contact-examination rates, high treatment abandonment rates, and low cure rates suggest that the city’s disease burden will not decrease without improved prevention and control strategies; better understanding of the factors associated with both transmission and treatment abandonment is needed. This paper provides new evidence to inform better TB control strategies. It analyzes the spatial and temporal patterns of TB incidence and treatment abandonment rates in Fortaleza from 2007 to 2014, and assesses how such patterns are predicted by social and demographic determinants.

## Methods

### Study setting

Fortaleza is the fifth-largest Brazilian city (roughly 2.6 million inhabitants), and the most-densely populated state capital (8220 inhabitants per km^2^). It has 119 bairros (neighborhoods) grouped into six districts with similar socioeconomic conditions. In 2010, the United Nations reported Fortaleza among the seven most unequal cities in the world, with an income Gini coefficient above 0.60 [15]. Fortaleza doubled its population over the past 35 years with limited urban planning; around 40% of the population currently lives in precarious settlements with substandard infrastructure [16].

### Data sources and variables

Data on TB cases in Fortaleza from years 2007 to 2014 were extracted from the Notifiable Diseases Information System (SINAN), which stores records of diseases of mandatory notification in Brazil [17]. Cases were geocoded to a residential location, and to the health center where they were notified and treated, based on patient home addresses entered in Google Earth 7.1. We successfully geocoded 96.4% of the cases, summarized them by bairro, and generated TB incidence rates for each bairro combining all 9 years of observation. Since some administrative boundaries changed between 2007 and 2014, we generated 117 bairros that were consistent across the whole period.

We also extracted data from SINAN to characterize patient socio-demographic and TB clinical characteristics, including: notification year; age; sex; race (Brazil has five exhaustive and exclusive racial categories: white, black, brown – or mixed descent, yellow – which includes Asian descent - and indigenous); education; pregnancy status; HIV test history; diabetes status; alcohol use; other conditions likely to aggravate TB; whether living in an institution; whether TB was likely acquired at work; TB site; baseline x-ray and skin test results; number of acid-fast bacilli (AFB) tests and cultures performed, and the number that were positive; whether DOT was recommended and whether it was maintained throughout treatment; prescribed TB drugs; home location; and treatment facility location.

Finally, we extracted information from SINAN on whether or not the TB case was considered as abandoned. Specifically, treatment abandonment for TB is considered after 30 days of non-attendance at a clinic once treatment has been started. The Ministry of Health recommends a home search/visit to try to persuade the patient to continue treatment. If the patient is not found or refuses TB treatment, the case is considered as abandonment, and recorded as such on SINAN.

In addition, we extracted bairro-level variables from the 2010 Population Census [18] to control for additional factors expected to be associated with TB incidence, including: population size and density; proportion of residents living in informal settlements; mean number of people per sleeping room; mean household size; mean monthly income; literacy rate; and proportion of households with access to services (electricity, piped water, garbage collection, sewerage). Lastly, we obtained information on reported AIDS cases from 2007 to 2013 from SINAN, and homicides from 2007 to 2014 from the Mortality Information System (as a proxy for neighborhood safety, which impacts access to healthcare, and contextual socioeconomic status, and thus public service provision) [10, 19].

### Statistical analyses

We conducted a spatial analysis of TB incidence rates in the 117 bairros grouped over 9 years. First, we conducted spatial mapping of TB rates and potential predictors across bairros. Second, we conducted Local Moran’s I analyses of bairro-level rates to identify clusters of significantly high or low TB rates, adjusting for multiple comparisons using the Benjamini-Hochberg method [20, 21]. Third, we ran conditional autoregressive (CAR) spatial regression models to identify factors associated with increased TB rates, accounting for spatial dependencies in the outcome. We ran bivariate models for each potential correlate of incidence rates, and then built a multivariable model containing all significant predictors from bivariate models, finally pruned back to contain only covariates significant at *α* = 0.05. CAR models had the general form:

$$ Log\left({\mu}_j\right)={E}_j+\sum_{k=1}^K{\beta}_k{X}_{k j}+\lambda {W}_j{u}_j+{\nu}_j $$

where the negative binomial of *μ*_*j*_ and *E* *j* are respectively the observed and expected TB case counts in each bairro summed across all 9 years, *X*_*kj*_ are *K* bairro-level covariates, and *W*_*j*_ is the spatial weights matrix generated using first-order Queen’s contiguity. Bairro-specific random effects are: *u*_*j*_, a spatial CAR Normally-distributed effect [22]; and *ν*_*j*_, a non-spatial log-normally distributed effect. Both random effects are modelled with mean 0 and variance *τ* ~ *gamma*(1, 0.026), such that 95% of residual incidence rate ratios fall between 0.5 and 2 [23]. All spatial analyses were conducted in R version 3.2 [24]; CAR models were conducted using Integrated Nested Laplace Approximation (INLA), a method that mimics Bayesian Monte Carlo Markov Chain (MCMC), using the INLA package [25].

To assess potential correlates of TB treatment abandonment we conducted a hierarchical analysis using two-level mixed-effect logistic regression models, allowing for random intercepts by bairro and random slopes by bairro over time, in a model of the form:

$$ {Y}_{ij}={\gamma}_{00}+{\gamma}_{0 k}{X}_{kij}+{\gamma}_{10} Tim{e}_{ij}+{\gamma}_{1 m}{Z}_{mj}+\left({\varepsilon}_{ij}+{\zeta}_{01}+{\zeta}_{10} Tim{e}_{ij}\right) $$

where *i* are individuals and *j* bairros, *γ*_*ij*_ are fixed effects, *ζ*_*ij*_ are random effects, *X*_*k*_ is a vector of individual-level covariates, and *Z*_*m*_ is a vector of bairro-level ones. *Time*_*ij*_ was measured as years since 2007. The model can be extended to allow for interactions between fixed effects, or for *X*_*k*_ or *Z*_*m*_ to vary across bairros. For these hierarchical models, we ran an unconditional means model, an unconditional growth model, and then bivariate models including *Time*_*ij*_. Finally, we built a multivariable model containing all variables significantly associated with treatment abandonment in the bivariate models, and pruned this model back to contain only significant covariates. These analyses were conducted in Stata 13 (StataCorp, College Station, TX). In all analyses, we used an indicator for missing covariate observations. The research protocol was approved by the Ethics Committee of the Federal University of Ceará.

## Results

Between 2007 and 2014, 12,352 primary cases of TB infection were reported. We dropped two observations that did not contain gender information, and 12 lacking geolocation, resulting in a working sample of 12,338 cases. Of those, 83.5% were pulmonary TB, 61.2% males, and 67.6% were cured (individual-level descriptive statistics are shown in Additional file 1).

Descriptive statistics for the sample at the bairro-level are provided in Table 1, and the spatial distribution of selected variables is shown in Additional file 2. At the bairro-level, public service provision was almost universal aside from sewerage; literacy was high, and mean household size relatively similar. Variations in population density, homicide rates, and AIDS case rates were more substantial. All bairro-level covariates, with the exception of electricity coverage, homicide rates, and AIDS case rates, tested significantly for positive spatial autocorrelation, and therefore were not homogenously distributed across the city.

**Table 1:** Bairro-level descriptive statistics and global Moran’s I for primary Tuberculosis cases in Fortaleza between 2007 and 2014

					Global Moran	
Variable	Unit	Median	IQR	SD	I	*p*-value
Proportion living in informal settlement		0.07	[0.00–0.17]	0.21	0.18	<0.001
Mean persons per sleeping room		3.45	[3.36–3.56]	0.19	0.52	<0.001
Mean monthly household income	Brazilian Reais (R$)	1821	[1352–2800]	1989	0.50	<0.001
Literacy	% of persons	94.28	[91.63–95.88]	3.11	0.39	<0.001
Electricity coverage	% of households	99.77	[99.64–99.87]	0.56	0.02	0.564
Water supply coverage	% of households	95.14	[91.05–97.00]	6.31	0.41	<0.001
Garbage collection coverage	% of households	99.64	[98.50–99.95]	3.35	0.13	0.005
Sewerage coverage	% of households	64.68	[24.92–89.09]	33.55	0.73	<0.001
Number of health posts (total of 92)		1.00	[0.00–1.00]	0.83	−0.01	0.998
Number of hospitals (total of 4)		0.00	[0.00–0.00]	0.89	0.13	0.004
Mean population 2007–14		137,084	[82,692–236,196]	117,282	0.18	<0.001
Population density 2007–14	per km2	11,309	[6178–15,031]	6161	0.55	<0.001
Homicide rate 2007–14	per 100,000 person-years	43.87	[21.09–72.36]	43.8	0.09	0.068
AIDS rate 2007–14	per 100,000 person-years	15.79	[6.25–37.64]	37.4	−0.01	0.964
Proportion of population white		0.37	[0.33–0.44]	0.10	0.35	<0.001
Proportion of population black		0.04	[0.03–0.06]	0.02	0.09	0.053
Proportion of population yellow		0.01	[0.01–0.02]	0.01	0.00	0.864
Proportion of population brown		0.57	[0.51–0.61]	0.15	0.25	<0.001
Proportion of population indigenous		<0.001	[0.00–0.00]	0.00	−0.01	0.992
TB case count 2007–14		80.00	[41.00–141.00]	88.0	0.30	<0.001
TB case rate 2007–14	per 100,000 person-years	8.21	[6.14–10.32]	4.33	0.26	<0.001
Abandonment count 2007–14		10.00	[4.00–22.00]	15.76	0.29	<0.001
Abandonment rate 2007–14	per 100,000 person-years	1.07	[0.70–1.62]	0.90	0.32	<0.001

*IQR* inter-quartile range, *SD* standard deviation, *I* Global Moran’s I statistic

**Fig. 1:**
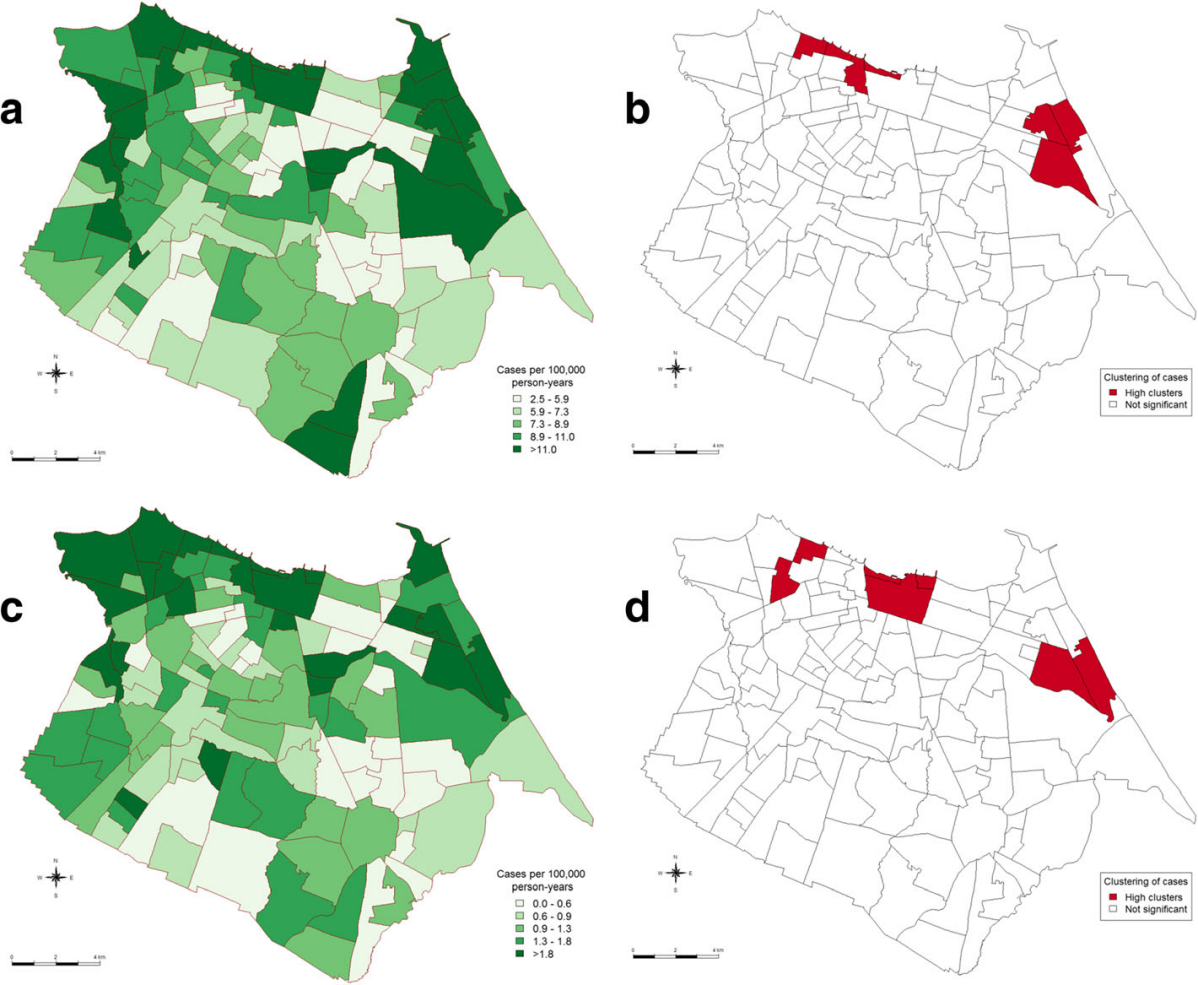
Bairro-level spatial descriptive statistics of Tuberculosis case rates in Fortaleza between 2007 and 2014. **a** Rates of newly notified TB cases; **b**. Local Moran’s I adjusted for multiple comparisons for newly notified TB case rates. **c** Rates of TB treatment abandonment; **d**. Local Moran’s I adjusted for multiple comparisons for TB treatment abandonment rates

The distribution of new and abandonment case rates are provided in Fig. 1. While TB cases were seen in all 117 bairros, case abandonment was not reported in two bairros. The local Moran’s I indicated that there were significant clusters of high incidence rates and abandonment rates in both the northwest and northeast of the city.

TB case rates were significantly spatially autocorrelated in a null model, with a global Moran’s I of 0.33; a spatial null model reduced this autocorrelation by two-thirds (Table 2). In bivariate spatial models, TB case rates were significantly higher in bairros with larger household sizes, lower income, lower literacy, less access to electricity, higher homicide rates, and larger proportion of self-reported black or brown residents. Bairros in District I had non-significantly higher case rates than elsewhere. In a multivariable model, bairros with higher literacy had lower TB rates, and those with higher sewerage access, higher homicide rates, and a greater proportion of self-reported black residents had higher TB rates. These four variables accounted for 74% of the spatial variance of the null spatial model, although there remained significant spatial autocorrelation within the multivariable model.

**Table 2:** Negative binomial spatial regression analyses of incident Tuberculosis case rates

Variable/Indicator	Null model	Null spatial model	Bivariate models		Multivariable model	
Log Population Density^a^			0.95	[0.85–1.06]		
Log Mean Monthly Income^a^			0.66	[0.57–0.77]		
Literacy rate			0.40	[0.22–0.72]	0.59	[0.44–0.80]
Mean household size			1.94	[1.25–3.03]		
Electricity connectivity			0.05	[0.01–0.17]		
Water supply			0.92	[0.80–1.07]		
Garbage collection			0.65	[0.50–0.85]		
Sewerage coverage			1.00	[0.97–1.04]	1.05	[1.02–1.08]
AIDS rate^b^			1.01	[0.93–1.10]		
Homicide rate^b^			1.16	[1.07–1.25]	1.08	[1.01–1.16]
Proportion of population white			0.87	[0.80–0.94]		
Proportion of population black			1.20	[1.12–1.30]	1.12	[1.03–1.21]
Proportion of population yellow			1.04	[0.96–1.12]		
Proportion of population brown			1.13	[1.04–1.23]		
Proportion of population indigenous			1.05	[0.97–1.13]		
District I			-	-		
District II			0.78	[0.51–1.18]		
District III			0.81	[0.58–1.13]		
District IV			0.72	[0.50–1.08]		
District V			0.72	[0.48–1.11]		
District VI			0.77	[0.51–1.24]		
Deviance Information Criterion	1137.3	1109.5			1065.5	
Spatial variance		0.117			0.031	
Non-spatial variance	0.025	0.015			0.012	
Spatial variance ratio		0.88			0.73	
Global Moran’s I	0.33	0.11			0.11	
Global Moran’s I *p*-value	0.00	0.03			0.03	

Values are incidence rate ratios and 95% confidence intervals associated with a 10 percentage point change in the value of each covariate, with the exceptions of log values (^a^: one log change), incidence rates (^b^: one standard deviation change) or indicators for District (difference relative to District I). Model diagnostics not shown for the 16 bivariate models; bivariate model for District contained five indicator variables

TB treatment abandonment occurred in 1901 cases, amounting to 15.4% of all new treatment episodes in Fortaleza between 2007 and 2014. Several variables were bivariately associated with treatment abandonment at both the individual- and bairro-level (Additional file 3). Table 3 shows the results of the multivariable analysis. The odds of abandonment showed an upward trend over the years, and in 2014 the odds of abandoning TB treatment were 71% higher than in 2007.

**Table 3:** Hierarchical multivariable logistic regression analysis of Tuberculosis treatment abandonment

Variable	Categories	Odds ratio	95% CI
Notification year (ref = 2007)	2008	0.98	[0.79–1.22]
	2009	1.05	[0.84–1.30]
	2010	1.15	[0.92–1.44]
	2011	1.39	[1.11–1.73]
	2012	1.17	[0.92–1.48]
	2013	1.42	[1.12–1.81]
	2014	1.71	[1.34–2.16]
Age (years; ref. = 0–9)	10–19	1.40	[0.71–2.75]
	20–29	1.95	[1.00–3.79]
	30–39	1.71	[0.88–3.32]
	40–49	1.30	[0.67–2.52]
	50–59	1.13	[0.58–2.22]
	60–69	0.71	[0.36–1.42]
	>69	0.65	[0.32–1.31]
Male		1.21	[1.08–1.35]
Race (ref = white)	Black	1.20	[0.95–1.52]
	Yellow	2.02	[1.31–3.11]
	Brown	1.28	[1.09–1.49]
	Indigenous	1.11	[0.46–2.73]
	Unknown	1.17	[0.91–1.49]
Education (ref = none)	Primary incomplete	0.90	[0.68–1.19]
	Primary complete	0.98	[0.73–1.33]
	Secondary incomplete	0.86	[0.65–1.14]
	Secondary complete	0.68	[0.49–0.93]
	High school incomplete	0.57	[0.41–0.79]
	High school complete	0.41	[0.29–0.57]
	College incomplete	0.29	[0.14–0.56]
	College complete	0.33	[0.19–0.59]
	Unknown	0.78	[0.60–1.02]
	Not applicable (age < 7)	0.75	[0.34–1.67]
Pregnant at diagnosis		0.47	[0.19–1.19]
HIV test (ref = negative)	Positive	2.10	[1.72–2.55]
	Not done	1.93	[1.71–2.16]
Alcohol use (ref = No)	Yes	1.76	[1.53–2.03]
	Unknown	1.26	[0.91–1.74]
Diabetes (ref = No)	Yes	0.59	[0.46–0.77]
	Unknown	0.84	[0.62–1.13]
Any other aggravating condition (ref = No)	Yes	1.39	[1.20–1.61]
	Unknown	0.89	[0.73–1.09]
TB type (ref = Pulmonary)	Extrapulmonary	0.74	[0.62–0.87]
	Both	0.74	[0.51–1.09]
First baseline culture (ref = Negative)	Positive	1.63	[1.15–2.31]
	Unknown	1.62	[1.20–2.19]
DOT recommended (ref = No)	Yes	1.43	[1.23–1.65]
	Unknown	1.10	[0.75–1.62]
DOT throughout treatment (ref = No)	Yes	0.69	[0.60–0.79]
	Unknown	0.74	[0.50–1.12]
Bairro-level literacy rate	10 percentage points	0.83	[0.61–1.13]
Bairro-level sewerage coverage	10 percentage points	1.05	[1.02–1.09]
Bairro-level: homicide rate	One standard deviation	0.92	[0.85–1.00]
District (ref = District I)	II	1.18	[0.90–1.56]
	III	0.69	[0.52–0.92]
	IV	0.91	[0.66–1.24]
	V	0.99	[0.73–1.34]
	VI	1.04	[0.77–1.40]
Constant		0.13	[0.01–2.20]
Bairro-level random effect variance		0.07	[0.03–0.12]

This model contained only covariates significant in bivariate models (results of bivariate models are shown in Additional file 3: Table S2). For bairro-level variables, odds ratios are those associated with a change in the covariate of the indicated amount

Variables that significantly increased the odds of abandonment in adjusted analysis included: later year of notification, being male, aged 20–29 (compared to ages 0–9), self-reported brown or yellow race (compared to white), HIV positive, culture positive at baseline; using alcohol, and having any other aggravating condition (e.g. tobacco use, other drug use, cardiovascular or lung conditions, cancer, malnutrition). Variables that significantly decreased the odds for treatment abandonment in adjusted analysis included: completed secondary education or more (compared to none), being pregnant, having diabetes, and having extra-pulmonary (compared to pulmonary) disease. Those who were recommended but did not complete DOT were more likely to abandon (odds ratio [OR]: 1.43, 95% confidence interval [CI]: 1.23–1.65), but those who remained on DOT throughout had lower odds of abandonment (OR: 0.69, 95%CI: 0.60–0.79).

Various TB drugs were associated with completion in bivariate analyses but none remained significant after accounting for other covariates. Living closer to the facility at which treatment took place was associated with lower odds of treatment abandonment, but at the median distance this amounted to only a 6% increased incidence rate. At the bairro-level, those living in areas with greater sewerage access and lower homicide rates had greater odds of abandonment.

## Discussion

Our results highlight several subtleties in the pattern and progression of TB in a relatively high-burden, high-inequality city. We found that TB case rates were clustered in areas with high rates of informal settlement and lower public service provision that lay relatively close to the center of the city. In multivariable analysis, after controlling for spatial autocorrelation, TB case rates were associated negatively with bairro-level literacy and positively with public sewerage provision, homicide rates and the proportion of population self-identifying as black. These findings coincide with other studies in Brazil showing case rates to be associated with low socioeconomic status at the area level [26–28].

Our results show that high case rates occur in geographically well-connected but unsafe and low socioeconomic status parts of the city. This suggests that publicly treated TB cases may over-represent accessible individuals, and point to the importance of active surveillance, including case finding and outreach [29], in the periphery of cities. These unstable peri-urban risk areas can be seen in many countries with high TB burdens [30, 31]. The current passive TB surveillance system, linked to mandatory reporting, has had a moderate impact on disease rates [32]. However, successful control and elimination of TB is likely to require a more active case-finding approach, focused on both high-risk settings and on contacts of index cases.

TB abandonment rates were clustered in similar areas to case rates, however, after adjusting for bairro-level literacy and sewerage access, bairro-level case rates were not significantly associated with risk of abandonment. In multivariable analysis, individual likelihood of abandonment was higher in men, those aged 20–40, those with lower education, and other conditions including HIV. Abandonment was also higher for those with pulmonary or culture-positive disease, and those who were recommended, but did not complete, DOT. These findings coincide with other studies showing abandonment to be associated with younger age within adulthood, lower socioeconomic status, alcohol use, HIV status and other comorbidities, and DOT [33, 34].

During the study period, DOT was recommended for approximately two-thirds of all new cases in Fortaleza (rising from 46% in 2007 to 87% in 2014), despite national guidelines recommending everyone receive DOT. Recommendation decisions could have been made informally by frontline staff based on perceived risk factors for treatment abandonment (e.g. alcoholism, retreatment after abandonment, homelessness, imprisonment and institutionalization [35]) and convenience (e.g. residence close to the clinic). DOT recommendation was associated with a 31% decrease in the odds of treatment abandonment amongst those who completed DOT. However, those recommended but not completing DOT had a 43% increased odds of abandonment, relative to those not recommended it. Predictors of being recommended DOT included younger age, lower education, using alcohol, living in District I and several factors associated with more transmissible disease – HIV negative, culture negative or having pulmonary disease (Additional file 3). Conditional on having started DOT, predictors of not completing DOT included self-identifying as brown, being HIV positive, having extrapulmonary disease, and not living in District I or IV. These associations suggest that those thought to be more infectious or at higher risk of failing treatment (e.g. pregnant women, extrapulmonary cases) are recommended to start DOT, and those with more complex cases (e.g. HIV positive) are less likely to complete. While pulmonary disease led to DOT being recommended more often (68% vs 56% for extrapulmonary disease), many potentially infectious individuals were not directed towards DOT. Thus, despite the increase in DOT recommendation rates, simply expanding DOT may be insufficient without investing additional resources in those incrementally covered.

The high rate of cases, but lower rates of abandonment in District I is also of interest. This area contains several older, large precarious settlements with high population density and low socioeconomic status. While these areas nominally have access to public services, the poorest households may not actually be served due to connection and usage fees. Additionally, the area has a long history of high TB rates, suggesting there may be substantial numbers of latent infections in the population. The well-established nature of the precarious settlements in this District means that healthcare provision here is somewhat stronger than that seen in other parts of the city. This combination of high incidence and low abandonment suggests that there is heterogeneity even within precarious areas, ensuring ongoing disease propagation: new infections are occurring in the better-served parts of the District (where most people are able to complete treatment), but undiagnosed cases continue to arise amongst the most marginalized, propagating the local epidemic.

### Strengths and limitations

One strength of this paper was the use of detailed administrative municipal TB records, which provide information on all individuals treated in the public-sector in Fortaleza. Some incident TB cases were treated privately and others not captured at all; nevertheless our sample represents the majority of the TB burden in the city. While there are no estimates for Fortaleza, only 13% of cases were estimated to be undiagnosed nationally in Brazil in 2015 [1]. One limitation is that bairro-level variables refer to 2010 only, and thus do not take account of any temporal changes over the 9 years of analysis. However, the variables included are unlikely to have changed greatly in this period, and 2010 is roughly the mid-point of our analytic period, somewhat limiting our concerns on this matter. Additionally, given the cross-sectional and ecological nature of all the exogenous variables considered in explaining TB incidence and abandonment, we are unable to tell if the associations shown are causal. This concern is lessened insofar as the goal of our analysis is to highlight predictors of poor outcomes, rather than causes.

Since Fortaleza represents a setting with relatively high TB risk and limited HIV co-infections, our results may be most applicable in other urban middle-income country settings. However, our key findings relating to spatial patterning of case finding and risk factors for abandonment should inform policy makers in many other urban settings. We highlight the importance of considering sociodemographic, behavioral and disease-specific factors when attempting to understand and mitigate the risk of infections and of treatment non-completion. While in Brazil such detailed information is collected through the regular health notification system, this is not the case for other TB endemic countries, hampering the possibility of better informing public policy. The benefits of such systems reinforce the importance of the full implementation of the broader DOTS strategy – including political commitment, quality case detection, supported treatment, effective drug supply, and careful monitoring and evaluation [36].

## Conclusion

Several social and demographic factors predict both neighborhood-level case rates and individual-level treatment abandonment. Yet, many are amenable either to direct intervention (e.g. by leveraging existing primary care services such as the FHS), or can be used to target enhanced case-finding or supervision to improve outcomes. Thus, there are possible avenues for action towards reducing local heterogeneities, and achieving the ambitious global targets to eliminate TB in the next 20 years.

## Additional files

Additional file 1: Table S1. Descriptive statistics for individual-level variables. (DOCX 30 kb)

Additional file 2: Figure S1. Choropleth maps of selected covariates. (DOCX 483 kb)

Additional file 3: Table S2. Hierarchical multivariable logistic regression analysis of: being recommended DOT (Model 1); abandoning DOT conditional on having been recommended DOT (Model 2). (DOCX 42 kb)

## Abbreviations

AFB: Acid-fact bacilli
CAR: Conditional autoregressive
CI: Confidence interval
DOT: Directly observed treatment
FHS: Family health strategy
HBC: High tuberculosis burden country
INLA: Integrated Nested Laplace Approximation
MCMC: Monte Carlo Markov Chain
MDG: Millennium development goals
OR: Odds ratio
SINAN: Notifiable Diseases Information System [Sistema de Informação de Agravos de Notificação]
TB: Tuberculosis
WHO: World Health Organization
