# Supplementary material for: Determinants of tuberculosis transmission and treatment abandonment in Fortaleza, Brazil
Source: BMC Public Health. 2017 May 25;17:508. doi: 10.1186/s12889-017-4435-0 (PMC5445312; doi:10.1186/s12889-017-4435-0)
Supplement: Supplementary file 1 — Descriptive statistics for individual-level variables. (DOCX 30 kb) [file 12889_2017_4435_MOESM1_ESM.docx]

## Additional File 1

**Title**: Determinants of Tuberculosis transmission and treatment abandonment in Fortaleza, Brazil

Table S1: Descriptive statistics for individual-level variables

| **Variable** | **Category** | **N** | **%** | **Median** | **IQR** |
| --- | --- | --- | --- | --- | --- |
| Notification year | 2007 | 1,442 | 11.69 |  |  |
|  | 2008 | 1,648 | 13.36 |  |  |
|  | 2009 | 1,744 | 14.14 |  |  |
|  | 2010 | 1,607 | 13.02 |  |  |
|  | 2011 | 1,559 | 12.64 |  |  |
|  | 2012 | 1,563 | 12.67 |  |  |
|  | 2013 | 1,466 | 11.88 |  |  |
|  | 2014 | 1,309 | 10.61 |  |  |
| Age | Years |  |  | 38 | [25 - 51] |
| Male^*^ |  | 7,551 | 61.20 |  |  |
| Race | White | 2,224 | 18.03 |  |  |
|  | Black | 862 | 6.99 |  |  |
|  | Yellow | 140 | 1.13 |  |  |
|  | Brown | 8,098 | 65.63 |  |  |
|  | Indigenous | 43 | 0.35 |  |  |
|  | Unknown | 971 | 7.87 |  |  |
| Education | Not applicable (age <7) | 267 | 2.16 |  |  |
|  | None | 485 | 3.93 |  |  |
|  | Primary incomplete | 1,813 | 14.69 |  |  |
|  | Primary complete | 857 | 6.95 |  |  |
|  | Secondary incomplete | 1,729 | 14.01 |  |  |
|  | Secondary complete | 856 | 6.94 |  |  |
|  | High school incomplete | 842 | 6.82 |  |  |
|  | High school complete | 1,169 | 9.47 |  |  |
|  | College incomplete | 163 | 1.32 |  |  |
|  | College complete | 280 | 2.27 |  |  |
|  | Unknown | 3,877 | 31.42 |  |  |
| Pregnant at diagnosis^*^ |  | 78 | 0.63 |  |  |
| HIV test result | Positive | 911 | 7.38 |  |  |
|  | Negative | 5,103 | 41.36 |  |  |
|  | Not conducted | 6,324 | 51.26 |  |  |
| Alcohol use^*^ | Yes | 1,693 | 13.72 |  |  |
|  | Unknown | 1,308 | 10.60 |  |  |
| Diabetes^*^ | Yes | 825 | 6.69 |  |  |
|  | Unknown | 1,526 | 12.37 |  |  |
| Any other aggravating condition^*^ | Yes | 1,672 | 13.55 |  |  |
|  | Unknown | 2,059 | 16.69 |  |  |
| Institutionalized | No | 10,637 | 86.2 |  |  |
|  | Prison | 218 | 1.77 |  |  |
|  | Elderly home | 15 | 0.12 |  |  |
|  | Orphanage | 39 | 0.32 |  |  |
|  | Psychiatric hospital | 30 | 0.24 |  |  |
|  | Other | 310 | 2.51 |  |  |
|  | Unknown | 1,089 | 8.83 |  |  |
| TB acquired at work^*^ | Yes | 307 | 2.49 |  |  |
|  | Unknown | 352 | 2.85 |  |  |
| TB type | Pulmonary | 10,301 | 83.49 |  |  |
|  | Extrapulmonary | 1,798 | 14.57 |  |  |
|  | Both | 239 | 1.94 |  |  |
| Baseline x-ray result | Suspect | 9,516 | 77.13 |  |  |
|  | Normal | 758 | 6.14 |  |  |
|  | Other pathology | 1,957 | 15.86 |  |  |
|  | Not conducted | 107 | 0.87 |  |  |
| Baseline skin test | Unreactive | 729 | 5.91 |  |  |
|  | Slightly reactive | 280 | 2.27 |  |  |
|  | Very reactive | 1,768 | 14.33 |  |  |
|  | Not conducted | 9,561 | 77.49 |  |  |
| Number of baseline AFBs |  |  |  | 2 | [1 - 2] |
| Number of positive baseline AFBs |  |  |  | 1 | [0 - 3] |
| First baseline culture | Positive | 909 | 7.37 |  |  |
|  | Unknown | 10,872 | 88.12 |  |  |
| Any other baseline culture^*^ | Positive | 287 | 2.33 |  |  |
|  | Unknown | 11,693 | 94.77 |  |  |
| Baseline histopathology test^*^ | AFB positive | 430 | 3.49 |  |  |
|  | Suggestive of TB | 685 | 5.55 |  |  |
|  | Not conducted | 11,094 | 89.92 |  |  |
| DOT recommended^*^ | Yes | 7,644 | 61.95 |  |  |
|  | Unknown | 725 | 5.88 |  |  |
| DOT throughout treatment^*^ | Yes | 4,775 | 38.70 |  |  |
|  | Unknown | 637 | 5.16 |  |  |
| Number of treatment AFBs |  |  |  | 1 | [0 - 3] |
| Rifampicin used^*^ | Yes | 11,839 | 95.96 |  |  |
|  | Unknown | 392 | 3.18 |  |  |
| Isoniazid used^*^ | Yes | 11,848 | 96.03 |  |  |
|  | Unknown | 388 | 3.14 |  |  |
| Ethambutol used^*^ | Yes | 6,759 | 54.78 |  |  |
|  | Unknown | 578 | 4.68 |  |  |
| Streptomycin used^*^ | Yes | 94 | 0.76 |  |  |
|  | Unknown | 801 | 6.49 |  |  |
| Pyrazinamide used^*^ | Yes | 11,692 | 94.76 |  |  |
|  | Unknown | 401 | 3.25 |  |  |
| Ethionamide used^*^ | Yes | 163 | 1.32 |  |  |
|  | Unknown | 805 | 6.52 |  |  |
| Other TB drug used^*^ | Yes | 222 | 1.80 |  |  |
|  | Unknown | 1,223 | 9.91 |  |  |
| Treated at nearest facility^*^ | Yes | 5,993 | 48.57 |  |  |
| Distance to treatment facility | km |  |  | 0.96 | [0.50 - 2.09] |
| Distance to notification facility | km |  |  | 0.98 | [0.51 - 2.22] |
| Distance to nearest facility | km |  |  | 0.60 | [0.37 - 0.90] |
| Final treatment outcome | Cured | 8,344 | 67.63 |  |  |
|  | Abandonment | 1,901 | 15.41 |  |  |
|  | Death, from TB | 406 | 3.29 |  |  |
|  | Death, not from TB | 279 | 2.26 |  |  |
|  | Transfer to other clinic | 1,014 | 8.22 |  |  |
|  | Change of diagnosis | 151 | 1.22 |  |  |
|  | Drug-resistance | 52 | 0.42 |  |  |
|  | Change of regimen | 1 | 0.01 |  |  |
|  | Primary abandonment | 1 | 0.01 |  |  |
|  | Continuing in treatment | 89 | 0.72 |  |  |
|  | Unknown | 100 | 0.81 |  |  |

N=12,338. For binary categorical variables (those marked^*^), negative responses are not shown, but account for all remaining observations.
