# Supplementary material for: Determinants of tuberculosis transmission and treatment abandonment in Fortaleza, Brazil
Source: BMC Public Health. 2017 May 25;17:508. doi: 10.1186/s12889-017-4435-0 (PMC5445312; doi:10.1186/s12889-017-4435-0)

## Additional File 2

**Title**: Determinants of Tuberculosis transmission and treatment abandonment in Fortaleza, Brazil

Figure S1: Choropleth maps of selected covariates

Location of areas of informal settlement
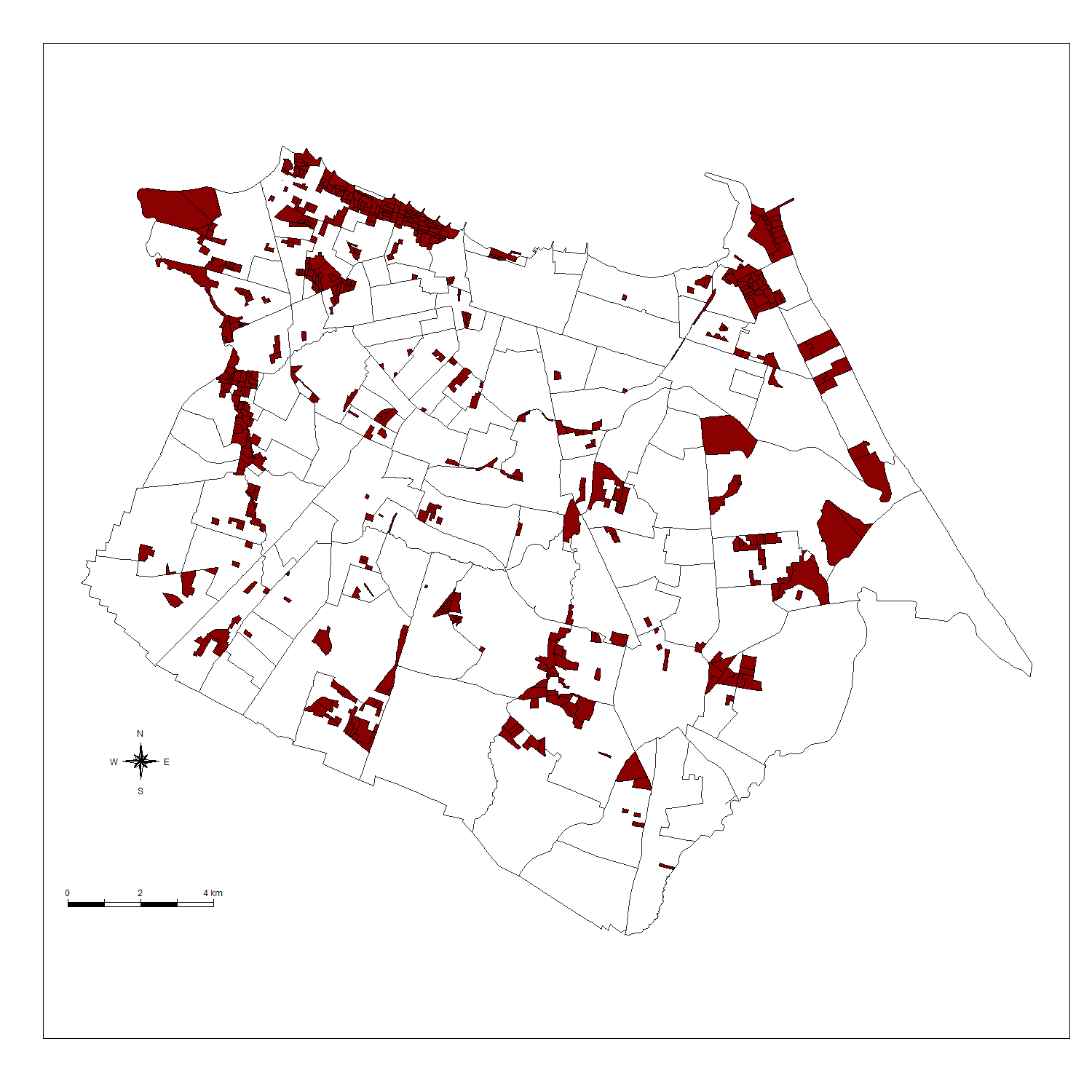


Average number of persons per sleeping room

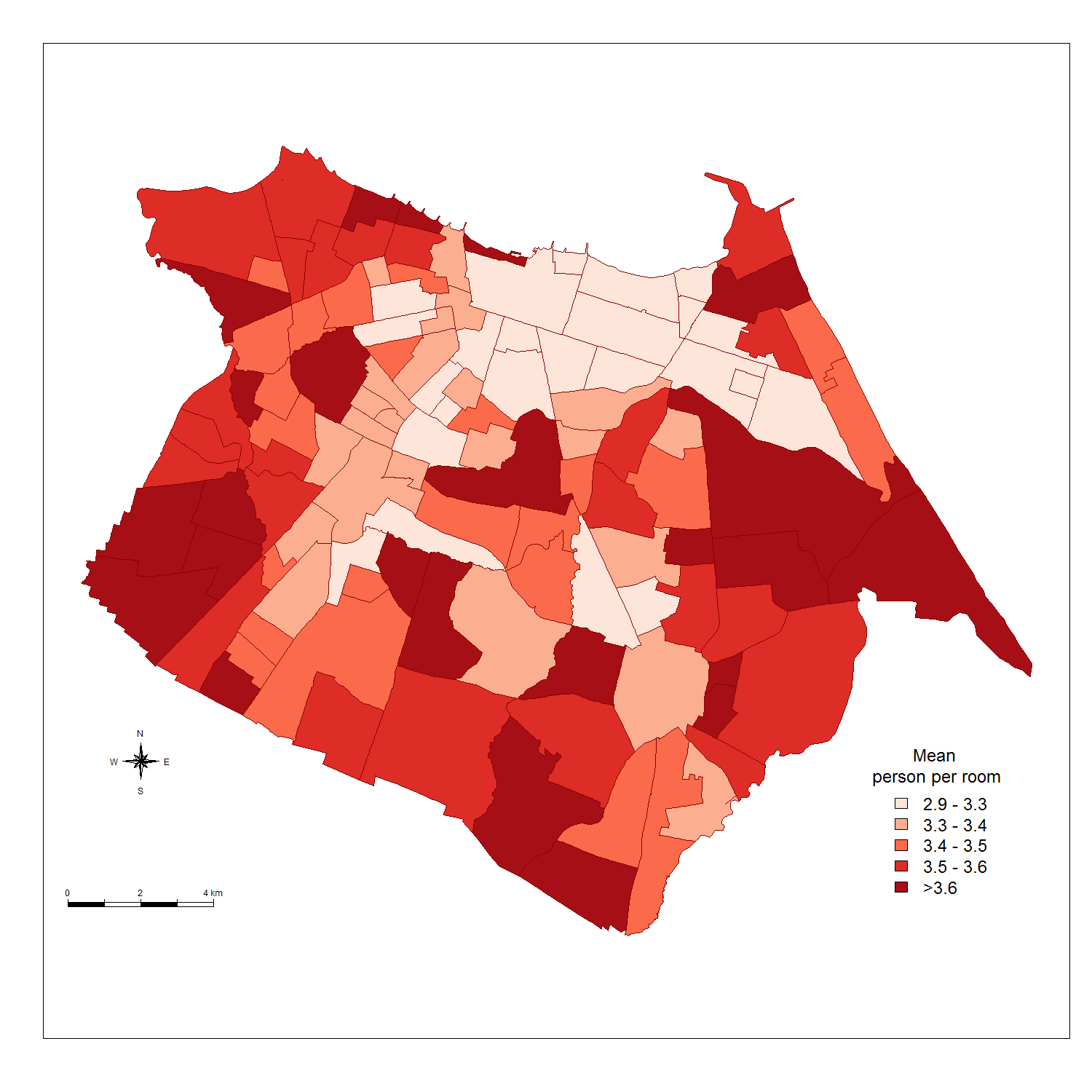


Mean monthly household income (R$)

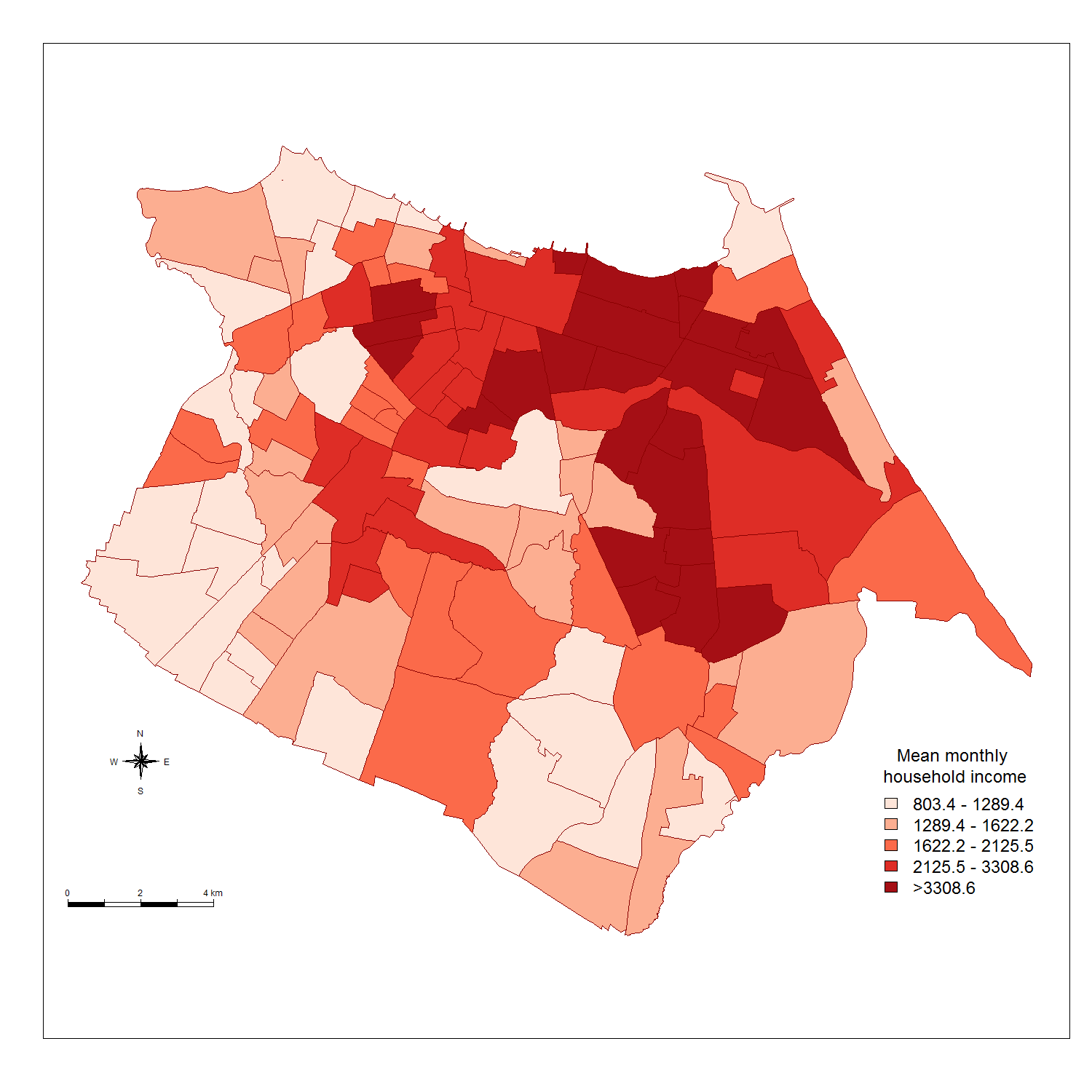


Literacy rate

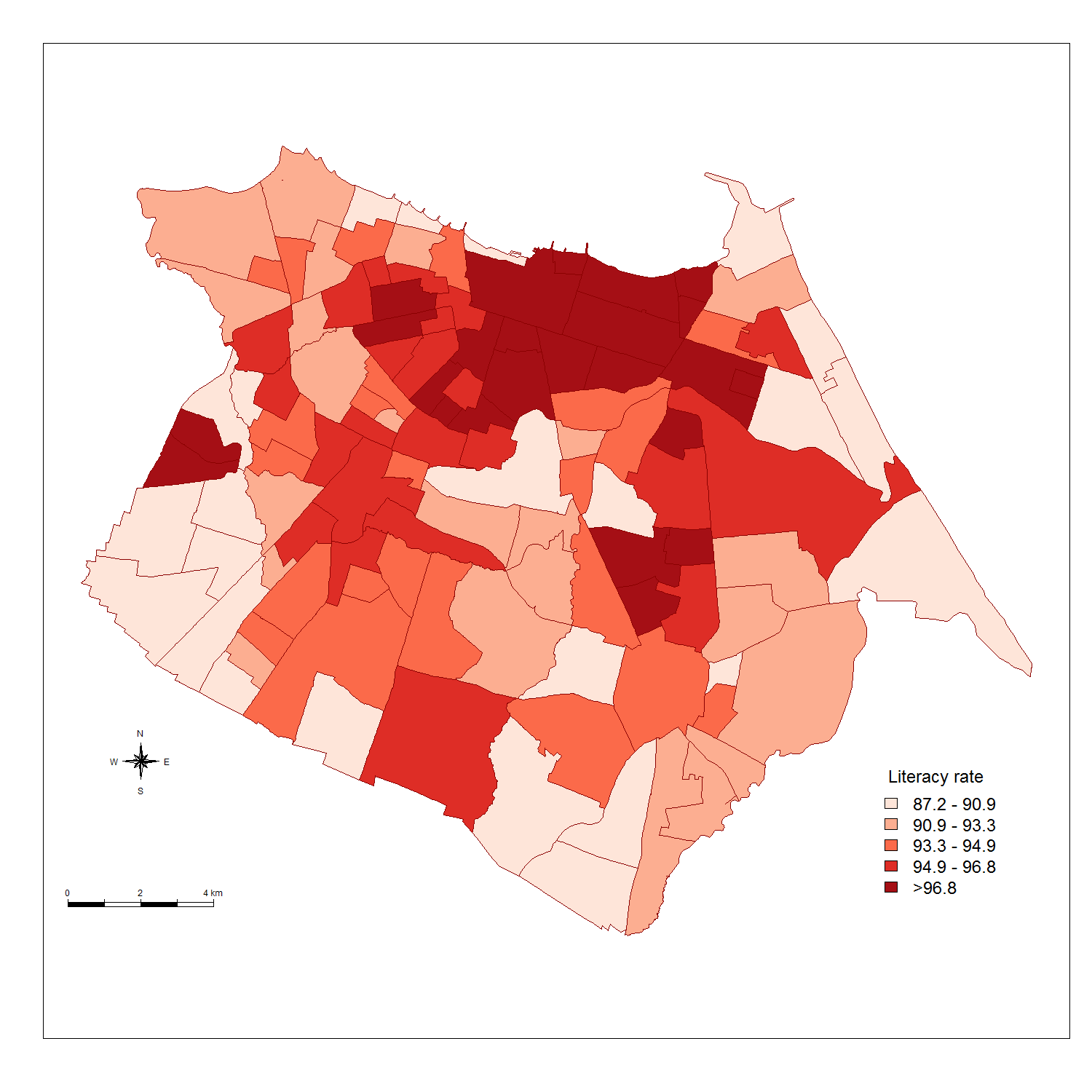


Percentage of households with access to electricity


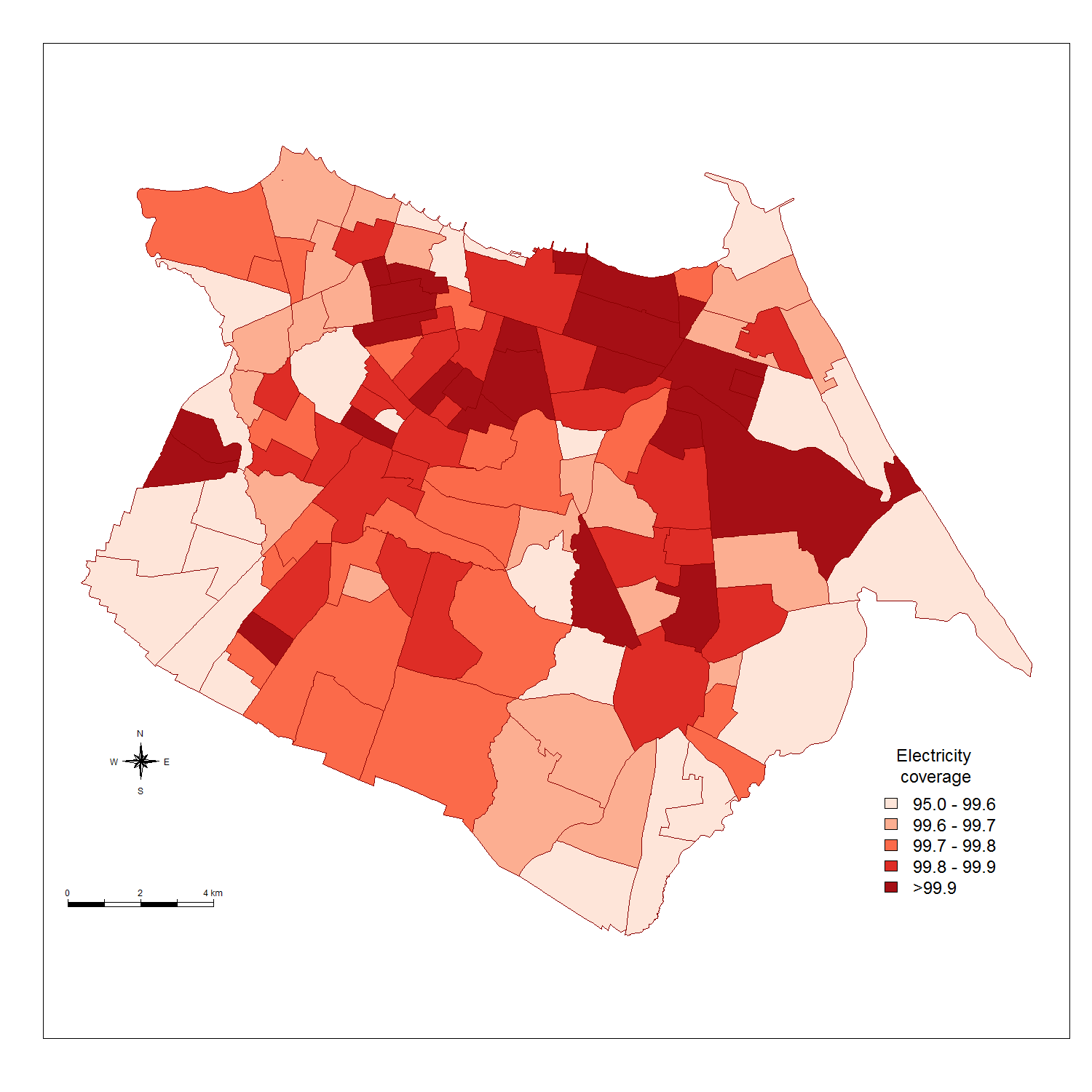


Percentage of households with access to piped water (%)

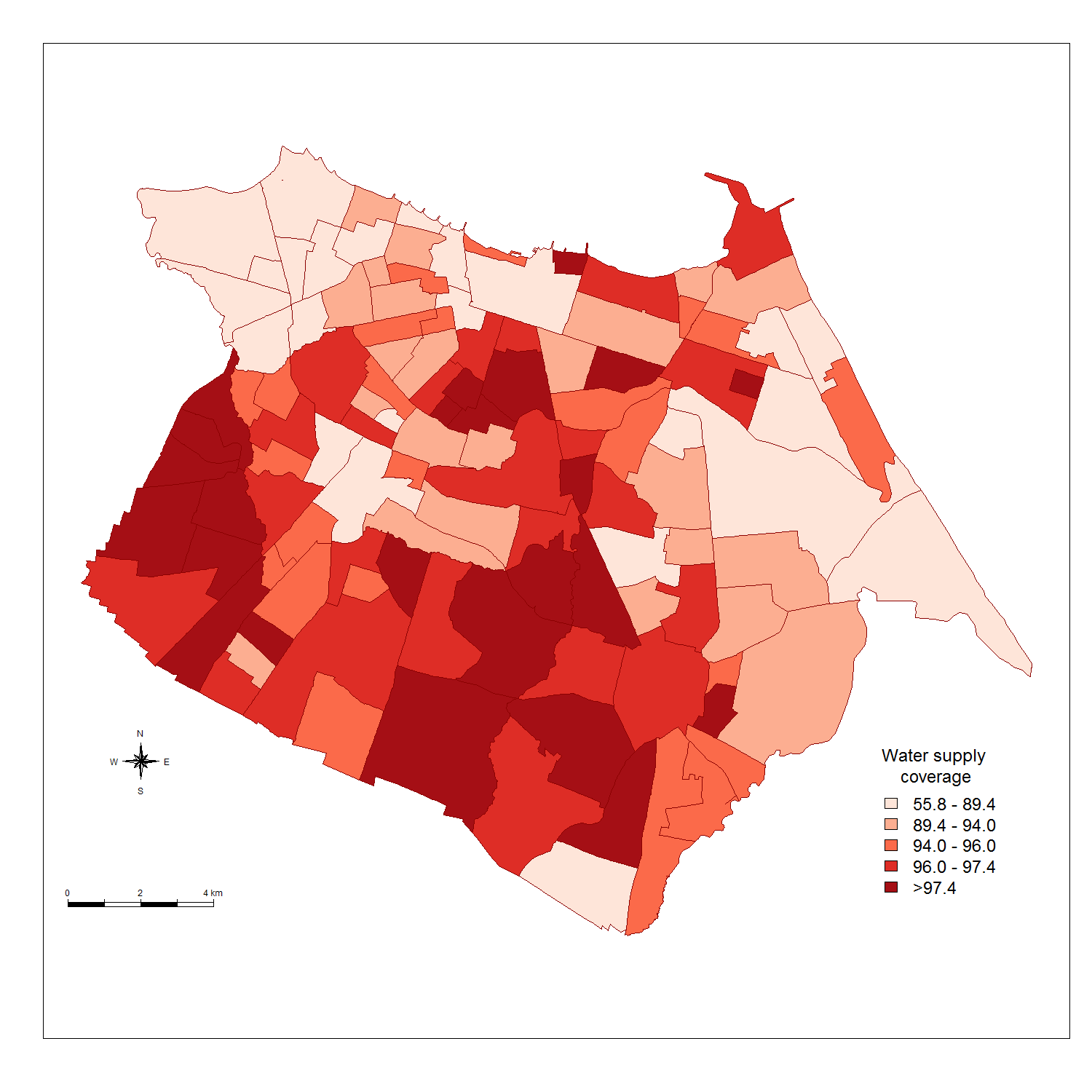


Percentage of households with public garbage collection (%)

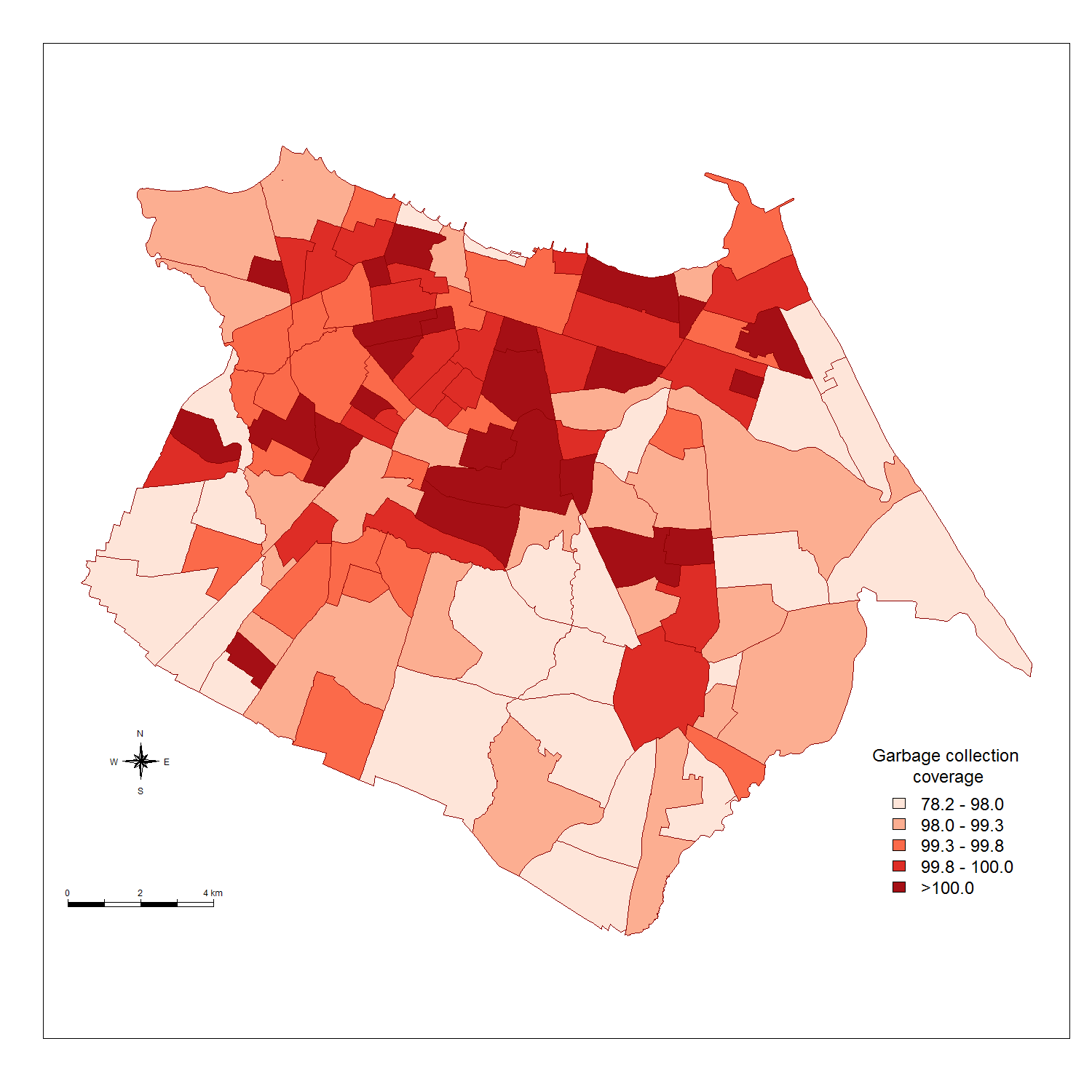


Percentage of households with access to public sewerage (%)


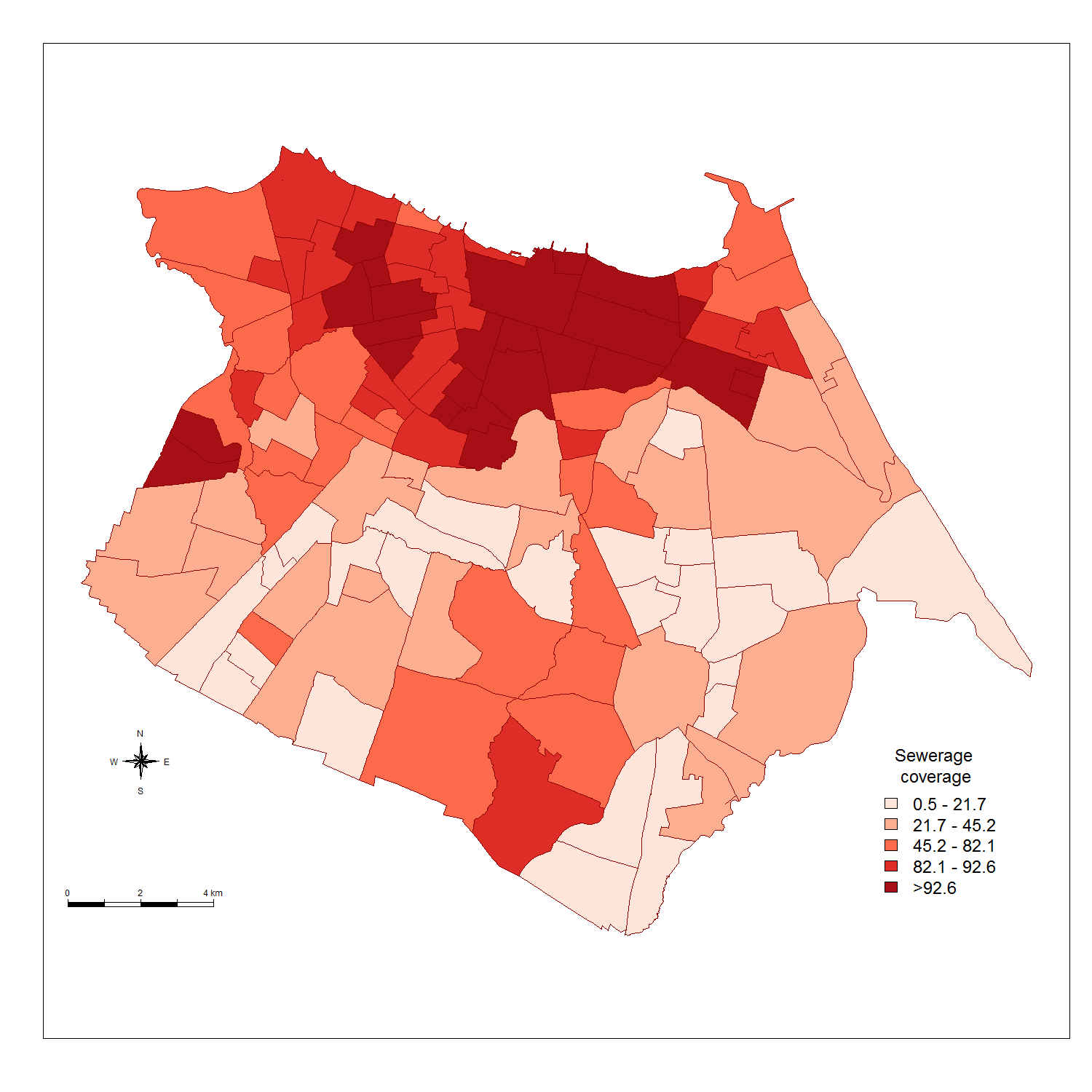


Population density (inhabitant/km^2^)

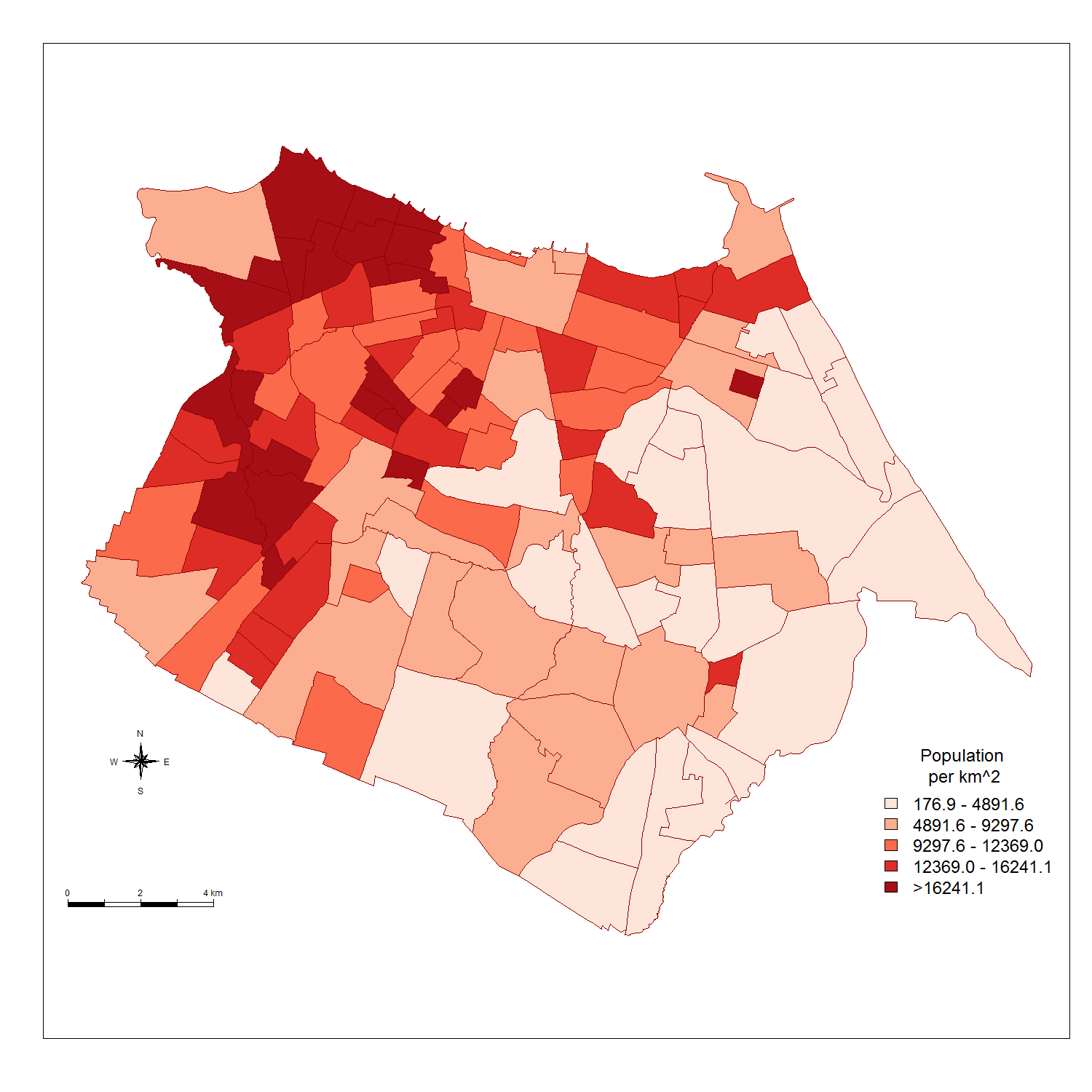


Homicide rate per 100,000 person-years


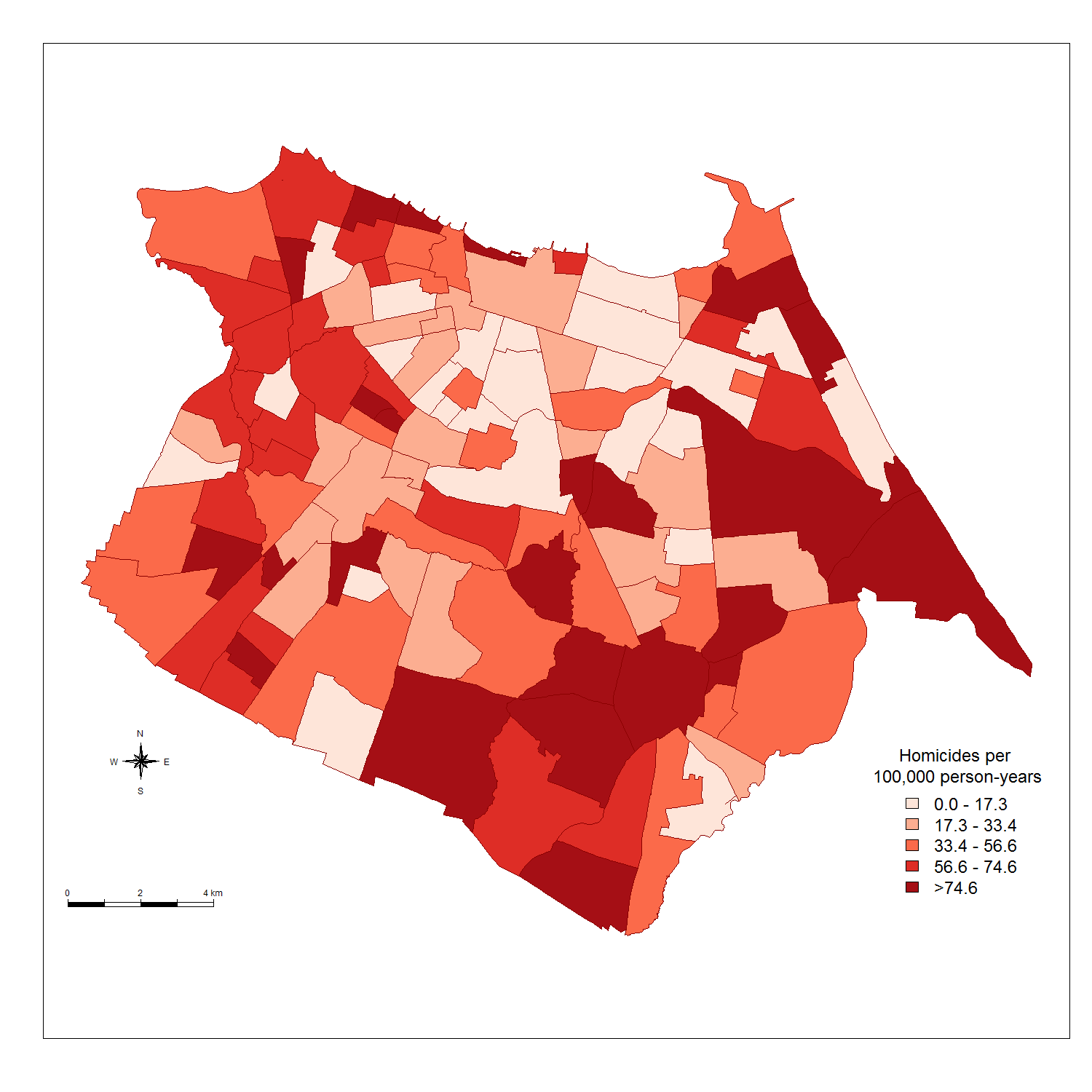


AIDS cases per 100,000 person-years


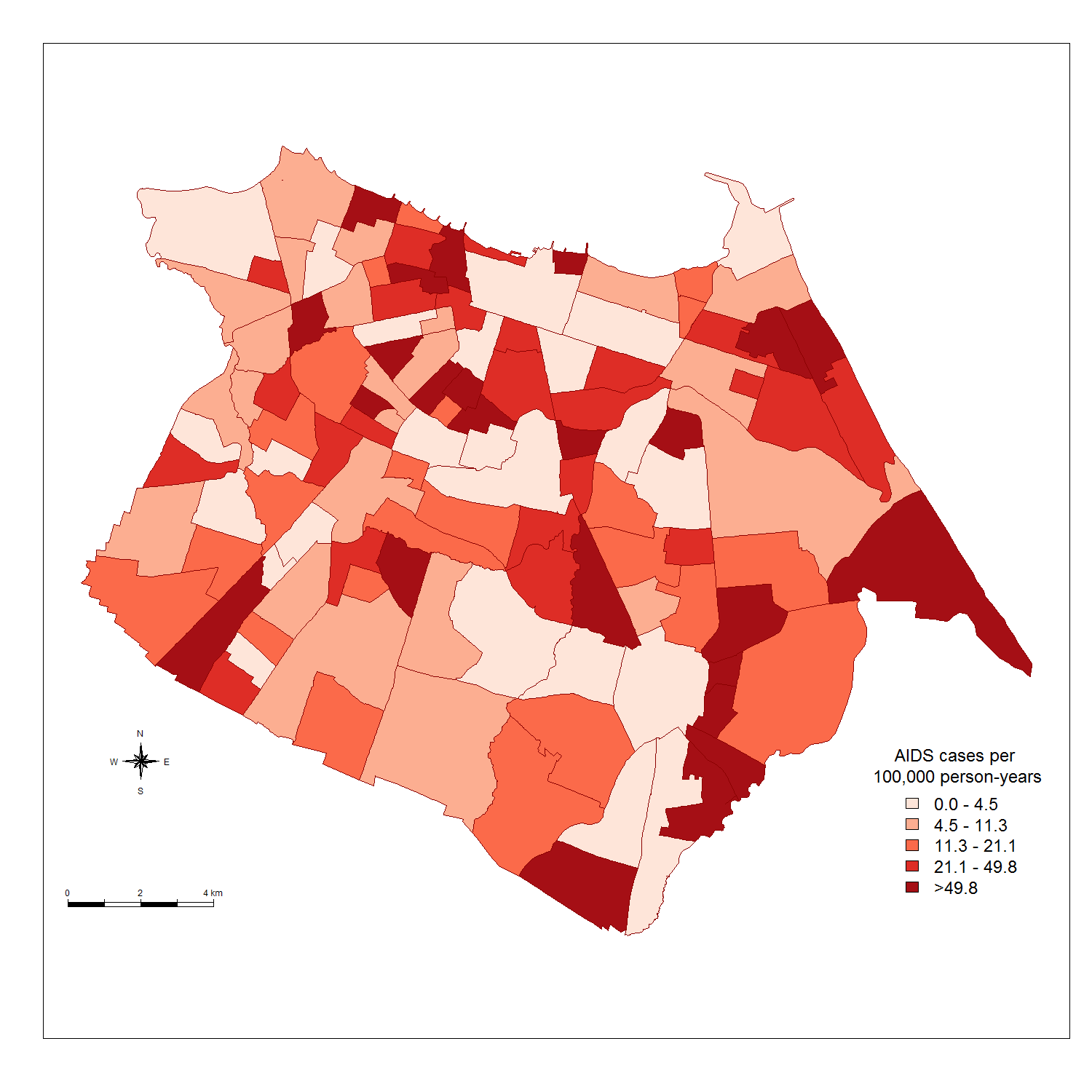

Supplement: Supplementary file 2 — Choropleth maps of selected covariates. (DOCX 483 kb) [file 12889_2017_4435_MOESM2_ESM.docx]
