# Supplementary material for: Determinants of tuberculosis transmission and treatment abandonment in Fortaleza, Brazil
Source: BMC Public Health. 2017 May 25;17:508. doi: 10.1186/s12889-017-4435-0 (PMC5445312; doi:10.1186/s12889-017-4435-0)
Supplement: Supplementary file 3 — Hierarchical multivariable logistic regression analysis of: being recommended DOT (Model 1); abandoning DOT conditional on having been recommended DOT (Model 2). (DOCX 42 kb) [file 12889_2017_4435_MOESM3_ESM.docx]

## Additional File 3

**Title**: Determinants of Tuberculosis transmission and treatment abandonment in Fortaleza, Brazil

Table S2: Hierarchical bivariate logistic regression analysis of Tuberculosis treatment abandonment

| **Variable** | **Category** | **Odds ratio** | **95% CI** |
| --- | --- | --- | --- |
| ***Individual-level variables*** |  |  |  |
| Notification year | 2007 | 1.00 |  |
|  | 2008 | 1.04 | [0.84 - 1.28] |
|  | 2009 | 1.07 | [0.87 - 1.32] |
|  | 2010 | 1.12 | [0.90 - 1.38] |
|  | 2011 | 1.44 | [1.18 - 1.77] |
|  | 2012 | 1.23 | [1.00 - 1.52] |
|  | 2013 | 1.50 | [1.22 - 1.84] |
|  | 2014 | 1.59 | [1.29 - 1.95] |
| Age (years) | 0-9 | 1.00 |  |
|  | 10-19 | 1.22 | [0.85 - 1.75] |
|  | 20-29 | 1.67 | [1.19 - 2.35] |
|  | 30-39 | 1.70 | [1.22 - 2.39] |
|  | 40-49 | 1.38 | [0.98 - 1.94] |
|  | 50-59 | 1.14 | [0.80 - 1.62] |
|  | 60-69 | 0.71 | [0.48 - 1.05] |
|  | >69 | 0.68 | [0.45 - 1.02] |
| Male |  | 1.45 | [1.30 - 1.61] |
| Race | White | 1.00 |  |
|  | Black | 1.61 | [1.29 - 2.02] |
|  | Yellow | 2.39 | [1.58 - 3.62] |
|  | Brown | 1.56 | [1.35 - 1.81] |
|  | Indigenous | 1.25 | [0.52 - 3.01] |
|  | Unknown | 1.25 | [0.99 - 1.57] |
| Education | None | 1.00 |  |
|  | Primary incomplete | 0.99 | [0.76 - 1.29] |
|  | Primary complete | 1.15 | [0.86 - 1.53] |
|  | Secondary incomplete | 1.08 | [0.83 - 1.41] |
|  | Secondary complete | 0.82 | [0.60 - 1.11] |
|  | High school incomplete | 0.68 | [0.50 - 0.93] |
|  | High school complete | 0.46 | [0.34 - 0.62] |
|  | College incomplete | 0.33 | [0.17 - 0.65] |
|  | College complete | 0.30 | [0.17 - 0.52] |
|  | Unknown | 0.90 | [0.70 - 1.15] |
|  | Not applicable (age <7) | 0.62 | [0.40 - 0.96] |
| Pregnant at diagnosis |  | 0.38 | [0.15 - 0.96] |
| Positive HIV test |  | 2.50 | [2.08 - 2.99] |
|  | Test not done | 1.87 | [1.67 - 2.09] |
| Alcohol use | Yes | 2.11 | [1.86 - 2.39] |
|  | Unknown | 0.98 | [0.83 - 1.17] |
| Diabetes | Yes | 0.51 | [0.40 - 0.65] |
|  | Unknown | 0.88 | [0.76 - 1.03] |
| Any other aggravating condition | Yes | 1.46 | [1.28 - 1.68] |
|  | Yes | 1.00 |  |
|  | Unknown | 0.87 | [0.75 - 1.00] |
| Institutionalized | No | 1.00 |  |
|  | Prison | 1.09 | [0.76 - 1.57] |
|  | Orphanage | 0.87 | [0.34 - 2.24] |
|  | Psychiatric hospital | 1.12 | [0.42 - 2.94] |
|  | Other | 1.12 | [0.83 - 1.53] |
|  | Unknown | 1.06 | [0.90 - 1.27] |
| TB acquired at work | No | 1.00 |  |
|  | Yes | 1.23 | [0.88 - 1.73] |
|  | Unknown | 1.01 | [0.69 - 1.46] |
| TB type | Pulmonary | 1.00 |  |
|  | Extrapulmonary | 0.67 | [0.58 - 0.79] |
|  | Both | 0.89 | [0.62 - 1.29] |
| Baseline x-ray | Suspect | 1.43 | [1.13 - 1.81] |
|  | Normal | 1.00 |  |
|  | Other pathology | 1.17 | [0.63 - 2.20] |
|  | Not conducted | 1.81 | [1.40 - 2.33] |
| Baseline skin test | Unreactive | 1.29 | [0.85 - 1.95] |
|  | Slightly reactive | 1.00 |  |
|  | Very reactive | 0.98 | [0.66 - 1.45] |
|  | Not conducted | 1.39 | [0.97 - 2.01] |
| Number of baseline AFBs |  | 0.96 | [0.91 - 1.02] |
| Number of positive baseline AFBs |  | 1.05 | [1.02 - 1.09] |
| Number of positive baseline AFBs | 0 | 1.00 |  |
|  | 1 | 1.14 | [0.99 - 1.31] |
|  | 2 | 0.73 | [0.43 - 1.25] |
|  | 3 | 1.20 | [1.07 - 1.34] |
|  | 4 | 1.01 | [0.69 - 1.48] |
| First baseline culture | Positive | 1.83 | [1.30 - 2.56] |
|  | Negative | 1.00 |  |
|  | Unknown | 1.77 | [1.32 - 2.37] |
| Any other baseline culture | Positive | 0.89 | [0.56 - 1.41] |
|  | Negative | 1.00 |  |
|  | Unknown | 1.08 | [0.80 - 1.47] |
| Baseline histopathology test | AFB positive | 0.71 | [0.41 - 1.24] |
|  | Suggestive of TB | 0.67 | [0.39 - 1.14] |
|  | Not suggestive of TB | 1.00 |  |
|  | Not conducted | 0.96 | [0.59 - 1.54] |
| DOT recommended | Yes | 1.28 | [1.14 - 1.43] |
|  | No | 1.00 |  |
|  | Unknown | 1.04 | [0.83 - 1.31] |
| DOT throughout treatment | Yes | 0.83 | [0.75 - 0.93] |
|  | No | 1.00 |  |
|  | Unknown | 0.77 | [0.61 - 0.98] |
| Number of treatment AFBs | 0 | 1.00 |  |
|  | 1 | 1.10 | [0.96 - 1.26] |
|  | 2 | 0.52 | [0.44 - 0.61] |
|  | 5 | 0.17 | [0.14 - 0.21] |
|  | 4 | 0.16 | [0.11 - 0.23] |
|  | 5 | 0.08 | [0.04 - 0.15] |
|  | 6 | 0.06 | [0.03 - 0.13] |
| Number of treatment AFBs |  | 1.04 | [0.95 - 1.14] |
| TB Drug: Rifampicin | Yes | 1.76 | [0.91 - 3.40] |
|  | No | 1.00 |  |
|  | Unknown | 1.29 | [0.63 - 2.66] |
| TB Drug: Isoniazid | Yes | 1.88 | [0.94 - 3.74] |
|  | No | 1.00 |  |
|  | Unknown | 1.38 | [0.65 - 2.94] |
| TB Drug: Ethambutol | Yes | 1.36 | [1.22 - 1.51] |
|  | No | 1.00 |  |
|  | Unknown | 0.93 | [0.71 - 1.21] |
| TB Drug: Streptomycin | Yes | 1.08 | [0.75 - 1.55] |
|  | No | 1.00 |  |
|  | Unknown | 0.81 | [0.51 - 1.30] |
| TB Drug: Pirazinamide | Yes | 1.24 | [0.83 - 1.86] |
|  | No | 1.00 |  |
|  | Unknown | 0.77 | [0.62 - 0.95] |
| TB Drug: Ethionamide | Yes | 0.88 | [0.49 - 1.59] |
|  | No | 1.00 |  |
|  | Unknown | 0.78 | [0.63 - 0.97] |
| TB Drug: Other | Yes | 0.87 | [0.59 - 1.28] |
|  | No | 1.00 |  |
|  | Unknown | 0.73 | [0.61 - 0.87] |
| Was treated at nearest facility |  | 0.99 | [0.89 - 1.09] |
| Distance to treatment facility | km | 1.06 | [1.01 - 1.12] |
|  | quadratic | 0.99 | [0.99 - 1.00] |
| Distance to notification facility | km | 1.07 | [1.01 - 1.13] |
|  | quadratic | 0.99 | [0.99 - 1.00] |
| Distance from household to nearest facility | km | 1.00 | [0.87 - 1.16] |
|  |  |  |  |
| ***Bairro-level variables*** |  |  |  |
| Proportion living in informal settlements | (10% points) | 1.09 | [0.97 - 1.22] |
|  | quadratic | 0.99 | [0.98 - 1.01] |
| No informal settlements |  | 0.94 | [0.76 - 1.16] |
| All informal settlements |  | 0.89 | [0.40 - 1.97] |
| Mean persons per sleeping room |  | 0.76 | [0.48 - 1.20] |
| Mean household size |  | 0.74 | [0.47 - 1.16] |
| Mean monthly household income | (R$ 1,000) | 0.89 | [0.74 - 1.06] |
|  | quadratic | 1.01 | [0.99 - 1.03] |
| Literacy rate^†^ | (10% points) | - | - |
|  | quadratic | - | - |
| Electricity coverage^†^ | (10% points) | - | - |
|  | quadratic | - | - |
| Water supply coverage | (10% points) | 1.56 | [0.32 - 7.56] |
|  | quadratic | 0.97 | [0.88 - 1.06] |
| Garbage collection coverage^†^ | (10% points) | - | - |
|  | quadratic | - | - |
| Sewerage coverage | (10% points) | 1.11 | [0.96 - 1.29] |
|  | quadratic | 0.99 | [0.98 - 1.01] |
| Number of health posts | 0 | 1.00 |  |
|  | 1 | 1.05 | [0.86 - 1.28] |
|  | 2 | 1.16 | [0.91 - 1.48] |
|  | 3 | 0.78 | [0.43 - 1.42] |
|  | 4 | 1.22 | [0.60 - 2.49] |
| Number of hospitals | 0 | 1.00 |  |
|  | 1 | 1.03 | [0.80 - 1.32] |
|  | 2 | 1.05 | [0.76 - 1.47] |
|  | 3 | 0.87 | [0.49 - 1.57] |
|  | 7 | 3.31 | [1.72 - 6.40] |
| TB case count in year |  | 1.00 | [0.99 - 1.01] |
| TB case rate in year | (One per 100,000 persons) | 1.00 | [1.00 - 1.00] |
| Population in year |  | 1.00 | [1.00 - 1.00] |
| Homicide rate in year | (One standard deviation) | 1.00 | [1.00 - 1.01] |
| Homicide rate in year | (One standard deviation) | 1.07 | [0.99 - 1.15] |
| AIDS rate in year | (One per 100,000 persons) | 1.00 | [0.99 - 1.02] |
| Population density in year | (One per km^2^) | 1.00 | [1.00 - 1.00] |
| TB case count in previous year |  | 1.01 | [1.00 - 1.01] |
| TB case rate in previous year | (One per 100,000 persons) | 1.00 | [1.00 - 1.00] |
| Abandonment count in previous year |  | 1.00 | [0.98 - 1.03] |
| Abandonment rate in previous year | (One per 100,000 persons) | 1.00 | [0.99 - 1.01] |
| Population in previous year |  | 1.00 | [1.00 - 1.00] |
| Homicide rate in previous year | (One per 100,000 persons) | 1.01 | [1.00 - 1.01] |
| AIDS rate in previous year | (One per 100,000 persons) | 1.00 | [0.99 - 1.02] |
| Population density in previous year | (One per km^2^) | 1.00 | [1.00 - 1.00] |
| District | I | 1.00 |  |
|  | II | 1.09 | [0.82 - 1.44] |
|  | III | 0.66 | [0.50 - 0.89] |
|  | IV | 0.82 | [0.61 - 1.09] |
|  | V | 0.79 | [0.60 - 1.05] |
|  | VI | 0.84 | [0.64 - 1.10] |

Coefficients in this table are from bivariate logistic models of each covariate against TB treatment abandonment, with spatial and non-spatial random intercepts at the bairro-level.

Values for continuous variables represent the effect of a change of indicated amount in the covariate of interest. Bivariate model for District contained five indicator variables.

^†^ Model did not converge.

Supplementary Table 2: Hierarchical multivariable logistic regression analysis of: being recommended DOT (Model 1); completing DOT conditional on having been recommended DOT (Model 2)

|  |  | **Model 1 (n=11,613)** | |  | **Model 2 (n=7,954)** | |
| --- | --- | --- | --- | --- | --- | --- |
| **Variable** | **Categories** | **Odds  ratio** | **95% CI** |  | **Odds ratio** | **95% CI** |
| Male |  | 1.08 | [0.98 - 1.18] |  | 1.06 | [0.95 - 1.18] |
| Notification year (ref=2007) | 2008 | 1.34 | [1.13 - 1.57] |  | 0.92 | [0.69 - 1.22] |
|  | 2009 | 1.52 | [1.29 - 1.79] |  | 0.88 | [0.66 - 1.16] |
|  | 2010 | 2.22 | [1.87 - 2.64] |  | 0.59 | [0.44 - 0.77] |
|  | 2011 | 2.35 | [1.98 - 2.80] |  | 0.22 | [0.17 - 0.28] |
|  | 2012 | 5.94 | [4.89 - 7.23] |  | 0.18 | [0.14 - 0.24] |
|  | 2013 | 9.51 | [7.63 - 11.85] |  | 0.20 | [0.15 - 0.26] |
|  | 2014 | 11.33 | [8.90 - 14.43] |  | 0.74 | [0.56 - 0.99] |
| Age (years; ref=0-9) | 10-19 | 0.46 | [0.25 - 0.83] |  | 0.73 | [0.39 - 1.35] |
|  | 20-29 | 0.41 | [0.23 - 0.73] |  | 0.88 | [0.48 - 1.62] |
|  | 30-39 | 0.37 | [0.21 - 0.67] |  | 0.86 | [0.47 - 1.59] |
|  | 40-49 | 0.45 | [0.25 - 0.80] |  | 0.94 | [0.51 - 1.73] |
|  | 50-59 | 0.37 | [0.20 - 0.67] |  | 0.93 | [0.50 - 1.72] |
|  | 60-69 | 0.43 | [0.23 - 0.78] |  | 0.86 | [0.46 - 1.61] |
|  | >69 | 0.42 | [0.23 - 0.78] |  | 1.00 | [0.53 - 1.88] |
| Race (ref=white) | Black | 0.92 | [0.76 - 1.11] |  | 1.23 | [0.96 - 1.57] |
|  | Yellow | 1.15 | [0.76 - 1.76] |  | 0.82 | [0.49 - 1.39] |
|  | Brown | 1.08 | [0.96 - 1.22] |  | 0.82 | [0.71 - 0.94] |
|  | Indigenous | 0.91 | [0.45 - 1.84] |  | 1.06 | [0.41 - 2.71] |
|  | Unknown | 0.94 | [0.77 - 1.14] |  | 1.76 | [1.34 - 2.31] |
| Education (ref=none) | Primary incomplete | 0.79 | [0.60 - 1.03] |  | 0.68 | [0.51 - 0.91] |
|  | Primary complete | 0.73 | [0.54 - 0.98] |  | 0.60 | [0.43 - 0.83] |
|  | Secondary incomplete | 0.80 | [0.60 - 1.05] |  | 0.77 | [0.57 - 1.04] |
|  | Secondary complete | 0.69 | [0.51 - 0.93] |  | 0.52 | [0.37 - 0.72] |
|  | High school incomplete | 0.63 | [0.46 - 0.84] |  | 0.76 | [0.54 - 1.06] |
|  | High school complete | 0.66 | [0.49 - 0.88] |  | 0.80 | [0.59 - 1.10] |
|  | College incomplete | 0.55 | [0.35 - 0.86] |  | 0.52 | [0.31 - 0.87] |
|  | College complete | 0.40 | [0.27 - 0.58] |  | 0.56 | [0.35 - 0.90] |
|  | Unknown | 0.60 | [0.46 - 0.77] |  | 0.50 | [0.38 - 0.66] |
|  | Not applicable (age <7) | 0.39 | [0.20 - 0.78] |  | 0.46 | [0.22 - 0.95] |
| Pregnant at diagnosis |  | 1.40 | [0.80 - 2.46] |  | 1.90 | [0.92 - 3.95] |
| HIV test (ref=negative) | Positive | 0.44 | [0.37 - 0.53] |  | 0.23 | [0.18 - 0.29] |
|  | Not done | 0.93 | [0.84 - 1.02] |  | 0.72 | [0.64 - 0.81] |
| Alcohol use (ref=No) | Yes | 1.35 | [1.17 - 1.55] |  | 1.09 | [0.93 - 1.27] |
|  | Unknown | 0.83 | [0.63 - 1.10] |  | 0.88 | [0.61 - 1.27] |
| Diabetes (ref=No) | Yes | 1.08 | [0.89 - 1.30] |  | 1.07 | [0.86 - 1.32] |
|  | Unknown | 1.25 | [0.96 - 1.62] |  | 0.90 | [0.65 - 1.26] |
| Any other aggravating condition (ref=No) | Yes | 1.06 | [0.93 - 1.22] |  | 1.10 | [0.94 - 1.28] |
|  | Unknown | 0.70 | [0.59 - 0.82] |  | 1.52 | [1.21 - 1.90] |
| First baseline culture (ref=Negative) | Positive | 1.41 | [1.08 - 1.85] |  | 1.08 | [0.80 - 1.46] |
|  | Unknown | 1.14 | [0.93 - 1.41] |  | 1.21 | [0.94 - 1.56] |
| TB type (ref-Pulmonary) | Extrapulmonary | 0.65 | [0.57 - 0.73] |  | 0.70 | [0.59 - 0.82] |
|  | Both | 0.76 | [0.55 - 1.04] |  | 0.49 | [0.32 - 0.73] |
| Bairro-level literacy rate | 10 percentage points | 0.91 | [0.66 - 1.25] |  | 1.14 | [0.78 - 1.66] |
| Bairro-level sewerage coverage | 10 percentage points | 0.99 | [0.95 - 1.02] |  | 0.95 | [0.91 - 0.99] |
| Bairro-level: homicide rate | One standard deviation | 0.99 | [0.90 - 1.08] |  | 1.03 | [0.93 - 1.13] |
| District (ref=District I) | II | 0.18 | [0.13 - 0.25] |  | 0.35 | [0.25 - 0.50] |
|  | III | 0.31 | [0.23 - 0.43] |  | 0.60 | [0.42 - 0.84] |
|  | IV | 0.36 | [0.26 - 0.50] |  | 0.19 | [0.13 - 0.28] |
|  | V | 0.58 | [0.42 - 0.81] |  | 0.60 | [0.41 - 0.87] |
|  | VI | 0.20 | [0.14 - 0.27] |  | 0.74 | [0.51 - 1.07] |
| Constant |  | 7.27 | [0.39 - 136.71] |  | 2.82 | [0.09 - 90.27] |
| Bairro-level random effect variance |  | 0.12 | [0.08 - 0.18] |  | 0.14 | [0.09 - 0.23] |
